# Predictors of severe hepatotoxicity among retroviral infected adults on HAART regimen in Ilubabor Zone, Southwest Ethiopia

**DOI:** 10.1038/s41598-024-57900-7

**Published:** 2024-04-11

**Authors:** Tefera Darge, Alemayehu Babusha, Dessalegn Chilo, Abebe Dukessa, Sisay Teferi

**Affiliations:** 1 Department of Biomedical Sciences, College of Health Sciences, Mettu University, Mettu, Ethiopia; 2 Department of Pharmacy, College of Health Sciences, Mettu University, Mettu, Ethiopia; 3 Department of Biomedical Sciences, Institution of Health, Jimma University, Jimma, Ethiopia; 4 Department of Medical Laboratory Science, College of Health Sciences, Mettu University, Mettu, Ethiopia

**Keywords:** Anti-retroviral therapy, Liver, Enzymes, Co-infection, Biochemistry, Physiology, Biomarkers

## Abstract

Nearly half of the deaths among hospitalized human immuno deficiency virus-infected patients in the highly active antiretroviral therapy era have been attributed to liver disease. This may range from an asymptomatic mild increase of liver enzymes to cirrhosis and liver failure. Different works of literature elucidated both retroviral infection and the adverse effects of highly active antiretroviral therapy as a cause of hepatotoxicity. Individual adaptations to medications and environmental exposures, shaped by cultural norms and genetic predispositions, could potentially modulate the risk and progression of liver disease in this population. Therefore, this study aims to assess the predictors of severe hepatotoxicity in retroviral-infected adults receiving highly active antiretroviral therapy regimens within the Ilubabor Zone, Southwest Ethiopia. A facility-based cross-sectional study was conducted among adult retroviral-infected patients in five selected anti-retro virus therapy clinics from May1 to July 30/2022. A systematic sampling technique was used to select 457 study participants and Binary logistic regression statistical data analysis was used, *P* value < 0.05 was considered statistically significant. The prevalence of severe hepatotoxicity was 21.44% in the study population. CD^+4^ count < 200 cells/mm^3^ (AOR = 2.19, 95% CI 1.04–5.22, *P* = 0.01), human immunodeficiency virus co-infection with tuberculosis (AOR = 2.82, 95% CI 1.01–8.29, *P* = 0.03) and human immuno deficiency virus co-infection with hepatitis-B/hepatitis C virus (AOR = 5.02, 95% CI 1.82–16.41) were predictors of severe hepatotoxicity. The magnitude of severe hepatotoxicity was high among adult retroviral-infected patients on highly active anti-retroviral drug regimens. Co-infection of human immuno deficiency virus with hepatitis B virus or hepatitis C virus, tuberculosis and CD4^+^T-cell count below 200 cells/mm^3^ were predictors of severe hepatotoxicity. Therefore, HIV patients on highly active antiretroviral therapy require close attention and regular monitoring of their liver function.

## Introduction

Human immunodeficiency virus (HIV) constitutes a significant global public health challenge of the twenty-first century. Untreated, HIV infection progressively weakens the immune system, rendering individuals highly susceptible to developing fatal opportunistic infections within a decade^1^. As of 2019, an estimated 38 million people worldwide were living with HIV, of whom 19.5 million had received antiretroviral therapy (ART)^2^.

Antiretroviral therapy (ART) involves using combinations of medications to manage HIV infection. Highly active antiretroviral therapy (HAART) is a specific type of ART that typically uses three or four drugs from different classes to maximally suppress the virus and reduce the risk of developing drug resistance, which can occur if the virus is not effectively controlled^2,3^. HAART plays a significant role in improving quality of life for individuals living with HIV. This is achieved through several mechanisms, including: reducing viral load, preserving and restoring the immune system, and decreasing both HIV-related morbidity and mortality^4^.

Liver disease in HIV/AIDS patients become a global concern as it affects a wide range of the population^5^. Since the widespread adoption of HAART medication, liver disorders have been implicated in over half of all deaths among hospitalized HIV-infected patients globally ^6^. The spectrum of liver disease in this population can range from asymptomatic mild elevations in liver enzymes to more serious conditions like cirrhosis and end-stage liver disease. Liver cirrhosis, a particularly severe consequence, carries an estimated overall prevalence of 8.3% in HIV-infected individuals^7^.

As the population of HIV-infected patients ages and remains on HAART for longer periods, hepatotoxicity due to HIV and HAART-related metabolic disorders has emerged as a significant public health problem^8^. Studies showed that the prevalence of liver enzyme elevation among HIV-positive individuals on ART ranged from 14 to 26.7%^7,9^.

The diagnosis of liver disease often relies on elevated levels of serum enzymes, particularly alanine aminotransferase (ALT), aspartate aminotransferase (AST), and alkaline phosphatase (ALP). These enzymes, involved in amino acid breakdown, serve as indicators of liver cell injury when their levels become abnormally high^4,9^.

Factors associated with hepatotoxicity in People living with HIV may be anti-retroviral and non-anti-retroviral drug-related toxicities; tumors (lymphoma and Kaposi sarcoma); and opportunistic infections such as cytomegalovirus or mycobacterium and co-infection with Hepatitis B virus (HBV) or Hepatitis C virus (HCV)^10,11^ Nucleoside reverse transcriptase inhibitors (NRTIs), protease inhibitors (PIs), and non-nucleotide reverse transcriptase are common causes of Anti-retroviral related hepatotoxicity .Other risk factors that contribute to liver disease are alcohol consumption, old age, female gender, and current CD4 < 200 cells/mm^3^^7^.

The presence of liver disease presents a significant complication to HIV management, leading to increased healthcare costs. It is also the leading cause for changes in or discontinuation of antiretroviral therapy, as well as medication non-adherence, all of which can ultimately result in treatment failure^8^. Therefore, comprehensive assessment of this toxicity and its associated factors is crucial to mitigating these potential problems. Different works of literature elucidated both retroviral infection and the adverse effects of HAART as a cause of hepatotoxicity. But these findings might be different in different geographical regions, culture and individual adaptations to various exposures. Therefore, this study is aimed to assess the predictors of severe hepatotoxicity in Retro Viral infected adults on HAART regimen in Ilubabor Zone Southwest Ethiopia.

## Methods and materials

### Study design

We conducted a facility based cross-sectional study design.

### Study Area

The study was conducted in the Ilubabor Zone ART center. Ilubabor Zone is found in the Oromia region at a distance of 625 km to the Southwest of Addis Ababa. There are 15 ART centers in Ilubabor Zone serving 2,791 HIV patients who are currently on HAART.

### Study Period

We carried out this study from June 1 to July 30/2022.

### Population

The source of the population for this study was all adult Retro viral infected patients who were on HAART drug regimen and attending their follow-up at ART clinics in Ilubabor Zone. The study population was all sampled adult retroviral-infected patients who were attending follow-up at the ART clinic and fulfilled the inclusion criteria.

### Inclusion and exclusion criteria

RVI patients on HAART and above 18 years old were included in this study while pregnant women, patients with liver disease before RVI, and Patients who are critically sick and unable to communicate were excluded from the study.

### Sample size determination and sampling techniques

For sample size determination we used a single population proportion formula, taking 95%CI, proportion 32% elevated Alanine Amin Transferase in Retroviral patients on HAART conducted in Bahir Dar^12^, and design effect of 1.5. As the source population was less than 10,000; sample size correction was performed. Then, a 10% non-response rate was added to obtain enough sample size.

*n* = (Z1- α/2)^2^ p (1 − *p*) = (1.96)^2^ X 0.32 (1–0.32) = 3.8416 × 0.32 × 0.68 = 334.

d^2^ (0.05)^2^ 0.0025.

Considering the design effect (1.5) = 334X1.5 = 501.

Using population correction formula nf = 501/1 + 501/2414 = 501/1.207 = 415.

Adding 10% non-response rate = 415 + 41.5 = 457.

### Sampling procedure

We used Simple random sampling and systematic sampling techniques respectively. There are fifteen ART centers in the Ilubabor Zone; five ART centers (Mettu karly comprehensive specialized Hospital, Darimu Hospital, Yayo, Hurumu and Gore Health Center) were selected by Simple random sampling technique. A systematic sampling technique was employed to selected ART centers after allocating the number of retroviral-infected patients on HAART to each ART center proportionally.

### Data collection procedures

We used interviewer-administered semi-structured questionnaires to collect Sociodemographic characteristics, clinical risk factors, behavior-related factors, and anthropometric data. We recruited five BSc nurses with previous experience in data collection and multilingual ability for data collection. The training was given to data collectors before the data collection period to address the objective of the study. Continuous follow-up and supervision were provided by the supervisor and principal investigator throughout the data collection periods.

### Anthropometric measurements

The height scale and the digital weighing machine were used to measure height and weight respectively. Subjects were weighed barefoot in very light clothing on digital weight scale and the measurement was recorded to the nearest decimal fraction. Height measurement was taken putting a person with feet flat, together, and against the vertical measuring board. Legs are straight, arms at sides, looking straight and posterior head touching vertical measuring board. Body mass index (BMI) was calculated as weight divided by the square of height in meters^13^.

Waist circumference was measured at the midpoint between the lower margin of the least palpable rib and the top of the hip or minimal waist using stretch-resistant tape while the subject stood with feet closed together thereby body weight evenly distributed arms at the side and wearing light clothing. When the subject became in a relaxed state measurement was taken at the end of normal expiration and this measurement was done in a private place^14^.

### Specimen collection, processing, and biochemical

We collected a 5-ml venous blood sample following an aseptic technique after the study participants held overnight fasting. Then we centrifuged the sample 30 min later at 3000 rpm for 10 min and stored the serum in a refrigerator at − 20 °C until use. Serum levels of liver enzyme biomarkers (ALT, ALP, and AST) were determined using an automated clinical chemistry analyzer COBAS-6000 (Germany), and viral hepatitis (HBV and HCV) were determined using a commercial test kit for HBSAg (Ameritech -China, Ltd., USA) and for anti-HCV (Wondfo Biotech Co., Ltd., Guangzhou, China)^15^.

### Operational definitions

Grade 1 hepatotoxicity: when an ALT value between 1.25 and < 2.5 × ULN.

Grade 2 hepatotoxicity: when an ALT value between 2.5 and < 5.0 × ULN.

Grade 3 hepatotoxicity: when an ALT value between 5.0 and < 10 × ULN.

Grade 4 hepatotoxicity: when ALT value ≥ 10 × ULN.

Severe hepatotoxicity is considered for grade 3 and grade 4 hepatotoxicity^16^

HAART Regimen:

Preferred first Line regimens: TDF + 3TC + EFV (FDC).

Alternative First Line regimens: AZT + 3TC + EFV, AZT + 3TC + NVP, TDF + 3TC + NVP and ABC + 3TC + EFV^17^.

### Data quality management and statistical analysis

All data were checked, cleared, and fed into Epi-data (version 3.1) and then exported to.

SPSS (version 25.0) software for statistical analysis. After the complete entry of all the data, a soft copy was checked with its hard copy to see the consistency. The data were also checked; for the fulfillment of the assumption. It was processed by using descriptive analysis, including frequency distribution. The association of independent variables with dependent variables was carried out using binary logistic regression. All independent variables with a *P* value < 0.25 in the bivariate logistic analysis were fitted into a multivariable logistic regression to identify independently associated factors in the final model. The degree of association was interpreted by using ORs with 95% CI and *P* < 0.05 was considered statistically significant. The Hosmer–Lemeshow test was used to check the appropriateness of the analysis model.

### Informed consent

The study protocol was evaluated and approved by the Institutional Review Board of Mettu University (Ref.No: RPG/135/2022), and ethical clearance was obtained. A formal letter was then requested from the Research and Postgraduate Coordinating Office of Health Science and presented to the medical directors of Darimu Primary Hospital, Mettu Karl Specialized Hospital, and the PHCU directors of Bure, Gore, and Yayo Health Centers. All methods were conducted in accordance with relevant guidelines and regulations. After thorough explanation of the study's objectives and purpose, written informed consent was obtained from all participants before data and sample collection. All data obtained during the study was kept confidential.

### Ethics approval and consent to participate

This study received ethical approval from Mettu University's Health Science College Research and Ethics Committee. All participants were voluntary and provided informed consent.

## Result

### Socio-demographic Characteristics

A total of 457 study participants were included in this study. Of these, 258 (56.46%) were females and the remainder were males. Participants were aged between 18 and 65 years, with a mean age of 46.74 ± 10.08 years. Regarding educational status, 198 (43.33%) had completed primary education, while only 80 (17.56%) had completed tertiary education (Table 1).

**Table 1:** Socio-demographic characteristics of adult retroviral infected patients on HAART in Ilubabor Zone, Mettu, 2022.

Variables	Category	Frequency	Percent
Age	< 40	122	26.69
	40–59	262	57.3 3
	≥ 60	73	15.97
Sex	Males	199	43.54
	Females	258	56.46
Educational status	No formal education	81	17.72
	Primary education	198	43.33
	Secondary education	98	21.44
	Tertiary education	80	17.51
Marital status	Single	41	8.97
	Married	209	45.73
	Divorced	108	23.63
	Widow	99	21.66
Monthly income	< 999	90	19.69
	1000–1999	111	24.29
	2000–2999	106	23.19
	≥ 3000	150	32.82

### Behavioral and clinical characteristics

Regarding behavioral characteristics, most individuals had no history of drinking alcohol (338, 73.96%) and were non-smokers (436, 95.40%). Regarding HAART duration, most patients stayed on HAART for more than 5 years (348, 76.15%).majority of the study participants no TB infection398 (87.09%) (Table 2).

**Table 2:** Behavioral and clinical characteristics of adult retro viral infected patients on HAART in Ilubabor Zone, Mettu, 2022.

Variables	Category	Frequency	Percentage (%)
Alcohol	Yes	119	26.04
	No	338	73.96
Smoking	Yes	21	4.59
	No	436	95.40
HAART duration(years)	$\le 5$	109	23.85
	> 5	348	76.15
Clinical Stage	I	233	50.92
	II	184	40.18
	III	40	8.89
TB co-infection	Yes	59	12.91
	No	398	87.09

**Figure 1:**
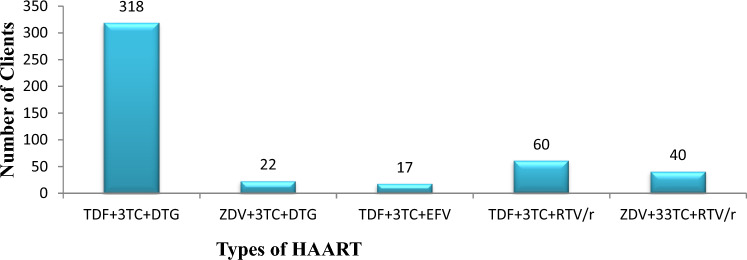
Types of HAART given for adult RVI patients in Ilubabor Zone, Southwest Ethiopia, 2022.

Concerning the type of HAART, the majority of clients were taking TDF + 3TC + DTG 318(69.58%) while only 17(3.72%) of them were taking TDF + 3TC + EFV Fig. 1.

### Anthropometric and biochemical characteristics

Approximately half of the study participants were in the normal range of their BMI (49.89%). Regarding liver enzymes, the majority of them had normal ALT (81.83%), AST (88.62%), and ALP (87.31%). The majority of the study participants were negative for both HBV (89.57%) and HCV (94.78%). Approximately half (48.58%) of the study participants had > 500 CD4 counts. The majority of the study participants with liver enzyme abnormalities had grade 1 hepatotoxicity (63.26%). (Table 3).

**Table 3:** Anthropometric and biochemical characteristics of adult retroviral infected patients on HAART in Ilubabor Zone, Mettu, 2022.

Variables	Category	Frequency (%)
BMI(Kg/m^2^)	Underweight	21 (4.59)
	Normal	228 (49.89)
	Overweight	139 (30.42)
	Obese	69(15.09)
ALT	Normal	374(81.83)
	Increased	83 (18.16)
AST	Normal	405 (88.62)
	Increased	52 (11.37)
ALP	Normal	399(87.31)
	Increased	58(12.69)
HBSAg	Negative	409 (89.49
	Positive	48 (10.50)
Anti-HCV	Negative	433 (94.75)
	Positive	24 (5.25)
Both HBsAg and Anti-HCV	Negative	431(94.31)
	Positive	26((5.69)
CD4^+^T^−^ cell count	< 200	95 (20.79)
	200–500	140(30.63)
	> 500	222 (48.58)
Hepatotoxicity	Grade 3	72 (15.75)
	Grade 4	26 (5.69)

BMI-Body mass index, ALT-Alanine Amino Transferase, AST-Aspartate Aminotransferase, Hepatitis B surface antigen, HCV-Hepatitis C virus, CD4-Cluster of Differentiation.

### Prevalence of severe hepatotoxicity

**Figure 2:**
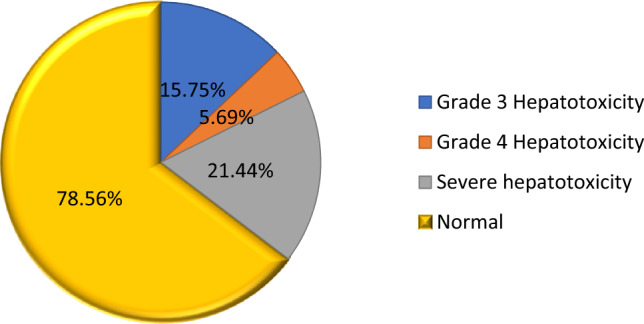
Prevalence of Hepatotoxicity among Retro Viral Infected Adults on Highly Active Anti Retro Viral Therapy in Ilubabor Zone, Southwest Ethiopia, 2022.

Ninety-eight (21.44%) of the study participants experienced severe hepatotoxicity for at least one biomarker (AST, ALP, or ALT). Elevations were observed in 83 (84.69%) for AST, 52 (53.06%) for ALT, and 36 (36.73%) for ALP. Notably, all three biomarkers (AST, ALT, and ALP) were elevated in the majority of participants (72, 15.75%), as shown in) (Fig. 2).

### Predictors of severe hepatotoxicity

A crude analysis was conducted to assess the presence of any association between the independent variables and severe hepatotoxicity. The following variables were selected for multivariable logistic regression analysis based on a *P* value threshold of less than 0.25: age, sex, CD4 level, BMI, TB-co-infection, duration on HAART, and viral hepatitis. After fitting into multivariable logistic regression analysis CD^+4^ count (AOR = 2.19, 95% CI 1.04–5.22), TB co-infection (AOR = 2.82, 95% CI 1.01–8.29) and HBV/HCV infection (AOR = 5.02, 95% CI 1.82–16.41 had a positive association with severe hepatotoxicity at *P* < 0.005 as shown in (Table 4).

**Table 4:** Multivariable logistic regression analysis of predictors of severe hepatotoxicity among adult retroviral infected patients on HAART in Ilubabor Zone, Mettu, 2022.

Variable	Category	Liver Enzyme		COR (95%CI)	AOR (95%CI)	*P* value
		Normal (%)	Abnormal (%)			
Age	< 40	98(21.44)	24(5.25)	1	1	
	40–59	219(47.92)	43(9.41)	0.80 (0.163–3.200)	0.85(0.12–3.01)	0.668
	≥ 60	42(9.19)	31(6.78)	3.01 (0.23–5.26)	2.54(0.2–3.5)	0.202
Sex	Male	146(31.94)	53(11.59)	1.92 (0.14–6.25)	2.01(0.74–7.56)	0.41
	Female	217(47.48)	41(8.97)	1	1	
CD^+4^ count cells/mm^3^	< 200	71(15.54)	24(5.25)	2. 25(1.02–5.12)	2.19 (1.04–5.22)*	0.01* 0.08
	200–500	123(26.91)	17(3.72)	0. 92 (0.21–3.34)	0.96 (0.28–3.41)	
	> 500	193(42.23)	29(6.35)	1	1	
BMI	Underweight	45(9.85)	18(3.94)	1.75 (0.44–3.46)	1.79(0.55–4.13)	0.32
	Normal	153(33.48)	35(7.66)	1	1	0.41
	Overweight	116(25.38)	22(4.81)	0.83(0.29 -2.38)	0.81(0.32–2.64)	0.17
	Obese	46(10.06)	22(4.81)	2.09(0.01–4.19)	2.5(0.2–4.77)	
TB co-infection	Yes	49(10.72)	10(2.18)	2.80 (0.67–7.25)	2.82(1.01–8.29)*	0.03*
	No	371(81.18)	27(5.91)	1	1	
Duration on	$\le 5$	76(16.63)	33(7.22)	1	1	
HAART	> 5	283(61.92	65(14.22)	0.53 (0.28–11.01)	0.63(0.3–12.22)	0.54
	Positive	55(12.03)	17(3.72)	4.45 (1.6 8–112.35)	5.02 (1.82–16.41)*	0.02*
HBV or HCV	Negative	360(78.77)	25(5.47)	1	1	

1-reference, COR-Crude Odd Ratio, AOR-Adjusted Odd Ratio, *P is significant at < 0.005, CD4-Cluster of differentiation, TB-Tuberculosis, HAART-Highly active anti-retroviral therapy, HBV-Hepatitis B virus, HCV-Hepatitis C virus.

E.g. Significant values are in [bold].

## Discussion

Hepatotoxicity is common in Retro Viral infected individuals and the degree of liver enzyme abnormality reflects the extent of liver injury. When there is an injury to the liver, variable concentrations of these enzymes are released into the blood due to increased permeability^18^. AST and ALT are frequently sensitive biomarkers of liver cell injury and are used for the detection of hepatocellular disorders. Different studies found that retroviral-infected patients on HAART had exhibited elevated levels of AST and ALT^9,19^.

The prevalence of severe hepatotoxicity in this study was 21.44% (98/457). This finding is in line with a study conducted at Debra Tabor in Ethiopia (20.1%), Cameroon (22.6%), and Brazil (19.7%)^20–22^. This may be attributable to the direct inflammation of hepatocytes by HIV through apoptosis, mitochondrial dysfunction, and permeability alteration in the mitochondrial membrane that stimulates an inflammatory response^23,24^.

However, our finding was lower than the study carried out in Eritrea and Bahirdar which reported severe hepatotoxicity s as 26.7% and 32% respectively^9,15^ . The discrepancy may be due to differences in environmental and genetic variation.

From this study, we found the association of severe hepatotoxicity with TB and HIV co-infection. Subjects with TB-HIV co-infection were 2 times (2.82, *P* = 0.03) more likely to acquire severe hepatotoxicity compared to Retroviral infected patients with no TB infection. This finding is consistent with different studies done elsewhere^25–27^*.* A possible explanation could be concomitant treatment with both anti-TB drugs and HAART which may exacerbate liver function derangement. Simultaneous administration of both HAART and anti-TB therapy might exacerbate the combined toxicity of both drugs. The possible mechanism for this enhanced toxicity is probably through induction and inhibition of enzymes necessary for Anti-retro viral drug metabolism secondary to complex drug interactions between ARV and anti-TB drugs^28,29^ .

This study also found that the current CD4 count < 200 cells/mm^3^ was significantly associated with severe hepatotoxicity. Patients with CD4 count < 200 were 2 times (AOR = 2.19, *P* = 0.01) more likely to develop severe hepatotoxicity compared to individuals with CD4 count > 500 cells/mm^3^. This finding was in agreement with other studies done in Kenya, Namibia, and Tanzania^30–32^. This might be because patients with low CD4 lymphocyte counts are more prone to acquiring opportunistic infections which might necessitate the consumption of different drugs leading to subclinical liver damage and thereby increased susceptibility for liver enzyme elevations while taking HAART^33^. Contrary to our finding, study conducted in Tanzania reported the association of high CD4 count (> 0.05 × 10^9^/L) with severe hepatotoxicity in adults retroviral-infected patients^34^. This can be explained by the difference in the study population as the study population for that study was HIV and Viral hepatitis entirely but most of our population was negative for viral hepatitis. Moreover, abnormal liver enzymes may be associated with immune reconstitution in case of high CD4 levels as that population was initiating HAART.

In our study, concomitant infection of the human immune deficiency virus with viral hepatitis (HBV or HCV) was significantly associated with severe hepatotoxicity. Study participants who had viral hepatitis infection were 5imes (AOR = 5.02, *P* = 0.02) more likely to develop severe hepatotoxicity compared to retroviral infected patients with no hepatitis virus infection. This finding is supported by other studies^35,36^.

Possible mechanisms of liver injury in these patients include an increase in the production of liver oxidative stress, mitochondrial injury, lipotoxicity, immune-mediated injury, cytotoxicity, toxic metabolite accumulation, systemic inflammation, senescence, and nodular regenerative hyperplasia^37,38^. Another possible scenario may be trough mutation in the HBV pre-core and overlapping core genes which is often associated with higher HBV DNA concentrations might contribute to severe hepatotoxicity in these co-infected patients^39,40^.

## Limitations of the study

The study has some limitations; first patients with elevated liver enzymes were not confirmed by more specific diagnostic tests like liver biopsy and ultrasound whether their liver was abnormal or not. Second, we used an anti-HCV antibody test which doesn't differentiate between active and previous infections as we couldn't use HCV RNA.

## Conclusions

This study found that retroviral-infected patients receiving HAART treatment were at increased risk for severe hepatotoxicity if they were co-infected with hepatitis B virus or hepatitis C virus, co-infected with tuberculosis or had a CD4^+^T-cell count below 200 cells/mm^3^. These findings underscore the importance of close monitoring of liver enzymes by clinicians for patients on HAART who present with these risk factors.

## Data Availability

The data sets used and/or analyzed during the current study are available from the corresponding author upon reasonable request.

## Abbreviations

AIDS: Acquired immunodeficiency syndrome
ALP: Alkaline phosphatase
ALT: Alanine amino transferase
ART: Anti-retro viral therapy
AST: Aspartate amino transferase
CD4: Cluster of differentiation4
HAART: Highly active anti-retroviral therapy
HBV: Hepatitis B virus
HCV: Hepatitis C virus
HIV: Human immunodeficiency virus
NRTI: Nucleoside reverse transcriptase inhibitors
NNRTI: Non-nucleoside reverse transcriptase inhibitors
PHCU: Primary health care unit
RNA: Ribonucleic acid
RVI: Retro viral infection

## References

[1] Wondifraw Baynes H, Tegene B, Gebremichael M, Birhane G, Kedir W, Biadgo B (2017). Assessment of the effect of antiretroviral therapy on renal and liver functions among HIV-infected patients: a retrospective study. HIV/AIDS Res. Palliat. Care.

[2] Osakunor DN, Obirikorang C, Fianu V, Asare I, Dakorah M (2015). Hepatic enzyme alterations in HIV patients on antiretroviral therapy: A case–control study in a hospital setting in Ghana. PLoS ONE.

[3] Fentahun A (2021). Antiretroviral treatment failure and associated factors among HIV-infected children on antiretroviral therapy: A retrospective study. HIV/AIDS Res. Palliat. Care.

[4] Ayelagbe OG, Akerele OP, Onuegbu AJ, Oparinde DP (2014). Drug hepatotoxicity in HIV patients on highly active antiretroviral therapy [HAART] in Southwest Nigeria. IOSR J. Dent. Med. Sci. (IOSR-JDMS).

[5] Yazie TS (2021). Derangement of liver enzymes, hyperglycemia, anemia, and associated factors among HIV-infected patients treated with tenofovir disoproxil fumarate-based regimen in Ethiopia: A prospective cohort study. Biomed Res. Int..

[6] Ezhilarasan D, Srilekha M, Raghu R (2017). HAART and hepatotoxicity. J. Appl. Pharm. Sci..

[7] Melashu Balew Shiferaw, Ketema Tafess Tulu AMZ and AAW. Liver Enzymes Abnormalities among Highly Active Antiretroviral Therapy Experienced and HAART Naïve HIV-1 Infected Patients at Debre Tabor Hospital, North West Ethiopia: A Comparative Cross-Sectional Study. Hindawi. 2016. 10.1155/2016/1985452 PMC4963552 27493798

[8] Bello SI, Onunu AN, Erah PO (2014). Long-term effect of HAART on biochemical profiles of HIV/AIDS patients in a tertiary health facility in Benin City. Trop. J. Pharm. Res..

[9] Achila, O. O., Kesete, Y., Abrhaley, F., Tesfaldet, F., Alazar, F., Fisshaye, L., Gebremeskel, L., Andemichael, D. & Mehari, R. Assessment of liver enzyme abnormality among HIV-infected patients on antiretroviral therapy in Asmara, Eriterea. *Res. Sq.* (2022). 10.1371/journal.pone.0270838 PMC9249179 35776747

[10] Llewellyn, A., Simmonds, M. & Brunton, G. Impact of antiretroviral therapy on liver disease progression and mortality in patients co-infected with HIV and hepatitis C: Systematic review and meta-analysis (2015). 10.1186/s41124-016-0015-7 PMC5918754 30288314

[11] Parameters of HIV-1 positive patients on antiretroviral therapy. *BMC* (2017).

[12] Wondemagegn M, Abera B (2013). Hepatotoxicity and associated risk factors in HIV-infected patients receiving antiretroviral therapy at Felege Hiwot referral hospital. Ethiop. J. Health Sci..

[13] Measuring Children's Height and Weight Accurately at Home (2021).

[14] Kefeni, B. T., Hajito, K. W. & Getnet, M. Renal function impairment and associated factors among adult HIV-positive patients attending antiretroviral therapy clinic in Mettu Karl referral hospital: A cross-sectional study. *Dove* (2021). 10.2147/HIV.S301748 PMC8200135 34135641

[15] Ezekiel OD (2018). Evaluation of the effects of anti-retroviral drugs on liver function tests of HIV-infected individuals. J. Med. Health Sci..

[16] Wondifraw Baynes H, Tegene B, Gebremichael M, Birhane G, Kedir W, Biadgo B (2017). Assessment of the effect of antiretroviral therapy on renal and liver functions among HIV-infected patients: A retrospective study. Dove.

[17] National Consolidated Guidelines for Comprehensive HIV Prevention, Care (2018).

[18] Qin F, Jiang J, Qin C, Huang Y, Liang B, Xu Y (2019). Liver Damage in patients living with HIV on antiretroviral treatment with normal baseline liver function and without HBV/HCV infection: An 11-year retrospective cohort study in. BMJ Open Diabetes Res. Care.

[19] Abongwa LE, Nyamache AK, Charles F, Torimiro J, Emmanuel N, Domkam I, Eyongetah M, Jude B, Mua FH, Bella S, Tamboh TC (2022). Risk factors for severe hepatotoxicity among HIV-1 infected individuals initiated on highly active antiretroviral therapy in the Northwest Region of Cameroon. BMC Gastroenterol..

[20] Bergsten-Mendes G (2014). Hepatotoxicity in HIV-infected children and adolescents on antiretroviral therapy. Sao Paulo Med. J..

[21] West N, Shiferaw MB, Tulu KT (2016). Liver enzyme abnormalities among highly active antiretroviral therapy experienced and HAART Naïve HIV-1 infected patients at Debre Tabor Hospital, North West Ethiopia: A comparative cross-sectional study. AIDS Res. Treat..

[22] Brumme, Z. Changes in mitochondrial DNA as a marker of nucleoside toxicity in HIV-infected patients. *ResGate* (2002). 10.1056/NEJMoa012035 11893792

[23] Stanislas Pol PL, Anaïs V-P (2004). HIV infection and hepatic enzyme abnormalities: Intricacies of the pathogenic mechanisms. CID.

[24] Enoh JE, Cho FN, Manfo FP, Ako SE, Akum EA (2020). Abnormal levels of liver enzymes and hepatotoxicity in HIV-positive, TB, and HIV/TB-coinfected patients on treatment in Fako division, Southwest Region of Cameroon. BioMed Res. Int..

[25] Gounder L, Moodley P, Drain PK, Hickey AJ, Moosa MS (2017). Hepatic tuberculosis in HIV co-infected adults: A case series of South African adults. BMC Infectious Dis..

[26] Naidoo K, Hassan-Moosa R, Mlotshwa P, Yende-Zuma N, Govender D, Padayatchi N (2020). High rates of drug-induced liver injury in people living with HIV coinfected with tuberculosis (TB) irrespective of antiretroviral therapy timing during antituberculosis treatment: Results from the starting antiretroviral therapy at three points in TB trial. Clin. Infectious Dis..

[27] Yimer G, Gry M, Amogne W, Makonnen E, Habtewold A (2014). Evaluation of patterns of liver toxicity in patients on antiretroviral and anti-tuberculosis drugs: A prospective four-arm observational study in Ethiopian patients. PloS ONE.

[28] Zeleke A, Misiker B, Yesuf TA (2020). Drug-induced hepatotoxicity among TB/HIV co-infected patients in a referral hospital. BMC Res. Notes.

[29] Opiyo WO, Ofulla AVO (2013). Liver function markers and associated serum electrolyte changes in HIV patients attending the patient support centre of Jaramogi Oginga Odinga teaching and referral hospital, Kisumu County, Kenya. East Afr. Med. J..

[30] Mugusi SF, Sando D, Mugusi FM, Hawkins C, Aboud S, Fawzi WW (2019). Risk factors for alanine aminotransferase elevations in a prospective cohort of HIV-infected Tanzanian adults initiating antiretroviral therapy. J. Int. Assoc. Provid. AIDS Care.

[31] Mataranyika PA, Kibuule D, Kalemeera F, Kaura H, Godman B, Rennie TW (2018). Liver enzyme elevations in a cohort of HIV/AIDS patients on first-line antiretroviral therapy in Namibia: Findings and implications. Alex. J. Med..

[32] Yimer G, Aderaye G, Amogne W, Makonnen E, Aklillu E, Yamuah L (2008). Anti-tuberculosis therapy-induced hepatotoxicity among Ethiopian HIV-positive and negative patients. PloS ONE.

[33] Sulkowski, M. S., Thomas, D. L., Chaisson, R. E. & Moore, R. D. Antiretroviral therapy in adults infected with human immunodeficiency virus and the role of hepatitis C or B virus infection. *Am. Med. Assoc.* (2010). 10.1001/jama.283.1.74 10632283

[34] Androutsakos T, Schina M, Pouliakis A, Kontos A, Sipsas N, Hatzis G (2020). Causative factors of liver fibrosis in HIV-infected patients. A single-center study. BMC Gastroenterol..

[35] Laguna-Meraz S, Roman S, Jose-Abrego A, Sigala-Arellano R, Panduro A (2022). Annals of hepatology: A hospital-based study of the prevalence of HBV, HCV, HIV, and liver disease among a low-income population in West Mexico. Ann. Hepatol..

[36] Kaspar MB, Sterling RK (2017). Mechanisms of liver disease in patients infected with HIV. BMJ Open Gastroenterol..

[37] Wong, V. W. S. & Chan, H. L. Y. 38. Hepatitis B and C in HIV/AIDS: Natural history of hepatitis co-infections in HIV/AIDS and effects of HIV on the treatment of chronic hepatitis B. *Am. J. Manag. Care* (2011).

[38] Suhail M, Abdel-Hafiz H, Ali A, Fatima K, Damanhouri GA, Azhar E (2014). Potential mechanisms of hepatitis B virus-induced liver injury. World J. Gastroenterol..

[39] Baseke J, Musenero M, Mayanja-Kizza H (2015). Prevalence of hepatitis B and C and relationship to liver damage in HIV-infected patients attending Joint Clinical Research Centre Clinic (JCRC), Kampala, Uganda. Afr. Health Sci..

[40] Revill PA, Littlejohn M, Ayres A, Yuen L, Colledge D, Bartholomeusz A (2007). Identification of a novel hepatitis B virus precore/core deletion mutant in HIV/hepatitis B virus co-infected individuals. AIDS.

